# Computational pharmacological comparison of *Salvia miltiorrhiza* and *Panax notoginseng* used in the therapy of cardiovascular diseases

**DOI:** 10.3892/etm.2013.1291

**Published:** 2013-09-10

**Authors:** CHUN-SONG ZHENG, XIAO-JIE XU, HONG-ZHI YE, GUANG-WEN WU, HUI-FENG XU, XI-HAI LI, SU-PING HUANG, XIAN-XIANG LIU

**Affiliations:** 1 Fujian Academy of Integrative Medicine, Fujian University of Traditional Chinese Medicine, Fuzhou, Fujian 350122;; 2 Fujian Key Laboratory of Integrative Medicine on Geriatrics, Fujian University of Traditional Chinese Medicine, Fuzhou, Fujian 350122;; 3 College of Chemistry and Molecular Engineering, Peking University, Beijing 100871, P.R. China

**Keywords:** *Salvia miltiorrhiza*, *Panax notoginseng*, cardiovascular disease, computational pharmacology

## Abstract

The herb pair comprising *Salvia miltiorrhiza* (SM) and *Panax notoginseng* (PN) has been used as a classical formula for cardiovascular diseases (CVDs) in China and in western countries. However, the pharmacology of SM and PN in this herb pair has not been fully elucidated. The aim of this study was to compare the mechanisms of SM and PN at the molecular level for the treatment of CVDs. We used a systems pharmacology approach, integrating ligand clustering, chemical space, docking simulation and network analysis, to investigate these two herbal medicines. The compounds in SM were attached to clusters 2, 3, 5, 6, 8 and 9, while the compounds in PN were attached to clusters 1, 2, 4, 5, 6, 7, 8 and 10. The distributions of chemical space between the compounds from SM and PN were discrete, with the existence of small portions of overlap, and the majority of the compounds did not violate ‘Lipinski’s rule of five’. Docking indicated that the average number of targets correlated with each compound in SM and PN were 5.0 and 3.6, respectively. The minority nodes in the SM and PN drug-target networks possessed common values of betweenness centrality, closeness centrality, topological coefficients and shortest path length. Furthermore, network analyses revealed that SM and PN exerted different modes of action between compounds and targets. These results suggest that the method of computational pharmacology is able to intuitively trace out the similarities and differences of two herbs and their interaction with targets from the molecular level, and that the combination of two herbs may extend their activities in different potential multidrug combination therapies for CVDs.

## Introduction

Cardiovascular diseases (CVDs) are the leading cause of death globally. Based on data from the World Health Organization (WHO), an estimated 17.3 million people died from CVDs in 2008, representing 30% of all global deaths (1). To date, the majority of patients with CVDs have been treated with drug therapies. The main drugs on the market are diuretics, vasodilators, anticoagulants, antiplatelet agents and β-blockers. While these drugs have yielded desired responses, they have also led to unwanted side-effects (2,3). Multiherb therapy, as an essential component of traditional medicine systems, has been utilized for thousands of years in China and other countries. It has exhibited improved curative efficacies and fewer side-effects, and has been used in the prevention of disease (4). It has been revealed that the majority of herbal medicines exhibit multiple cardiovascular effects (5). Thus, multiherb therapy may be one of the best conventional and complementary medical approaches in the prevention and treatment of CVDs.

*Salvia miltiorrhiza* (SM) and *Panax notoginseng* (PN) have been widely used in combination in Traditional Chinese Medicine (TCM) for the therapy of CVDs in China and other countries, including the United States (6–8). It has been demonstrated that these two herbs are compatible and have a synergistic effect (7). However, the molecular mechanisms underlying their compatibility have yet to be clearly elucidated. Numerous computational pharmacological studies, which have been generated using library analysis, quantitative structure-activity relationship (QSAR), receptor-ligand interaction and biological networks, have been developed to clarify the pharmacology and efficacy of TCM (9,10). Therefore, in the present study, we compared the computational pharmacology of SM and PN at the molecular level, in order to enhance the understanding of factors affecting compatibility in TCM and to accelerate modern TCM development.

## Materials and methods

### Preparation of SM and PN chemical databases

The structures identified in the medicinal herbs of SM and PN were taken from the Chinese Herbal Drug Database and the Handbook of the Constituents in Chinese Herb Original Plants (11,12). The total number of compounds in SM and PN was 53 and 57, respectively. These compounds were converted into three-dimensional structures and energy optimizations were performed using the Discovery Studio 2.0 (DS 2.0) software (Accelrys Inc., San Diego, CA, USA), based on the Merck Molecular Force Field (MMFF). Following this, the protocol of Cluster Ligands was used to cluster the compounds from the SM and PN chemical databases (13).

### Calculation of molecular descriptors

The protocol from ‘Calculate Molecular Properties’ in the QSAR module of DS 2.0 was employed to calculate the descriptors for the compounds from the SM and PN chemical databases. The chemical space was constructed using 150 diversity descriptors, including the molecular properties of one, two and three dimensions (14,15). Principal component analysis (PCA) was then performed to map the distribution of the compounds in chemical space.

### Molecular docking

The modern docking program LigandFit, within DS 2.0, was used to perform the molecular docking. The crystal structures of 16 key proteins associated with CVDs (16,17) were downloaded from the Research Collaboratory for Structural Bioinformatics (RCSB) protein data bank (PDB; Table I; www.rcsb.org). All crystallographic water was removed from the file and hydrogen atoms were added. The inhibitor from the PDB file was used to define the active site. The compounds from the SM and PN chemical databases were docked into the protein models. All docked structures were sorted according to their DockScore. The compounds with the top-five DockScores were selected as potential active compounds, as described previously (18).

### Network construction and analysis

Cytoscape 2.8.3 was used for network construction (19). The potential active compounds and their corresponding target proteins were connected to each other to generate a drug-target (D-T) network. In this network, the nodes represented compounds or proteins and the edges represented the compound-target interactions. All data were analyzed using Cytoscape plugins.

## Results

### Comparison of the SM and PN chemical databases: Clustering distribution

**Figure 1.**
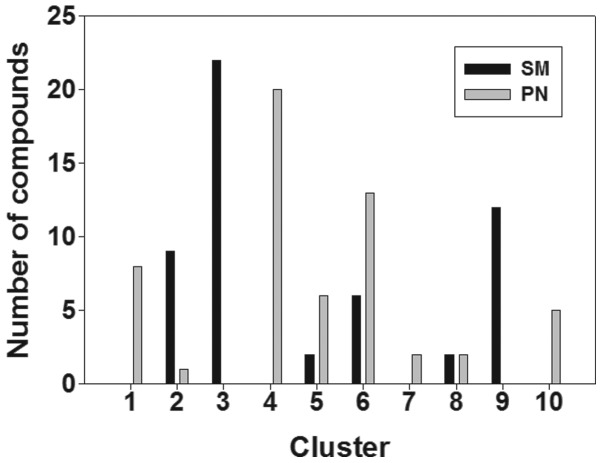
Clustering distribution of compounds from the chemical databases of *Salvia miltiorrhiza* (SM) and *Panax notoginseng* (PN).

The compounds from the SM and PN chemical databases were clustered by employing the default settings of Cluster Ligands (Fig. 1). Fig. 1 shows that the compounds in SM were attached to six clusters, known as clusters 2, 3, 5, 6, 8 and 9, while the compounds in PN were attached to eight clusters, known as clusters 1, 2, 4, 5, 6, 7, 8 and 10. These results indicate that SM and PN have similarities and differences with regard to chemical structure clustering.

### Comparison of the SM and PN chemical databases: Chemical space

**Figure 2.**
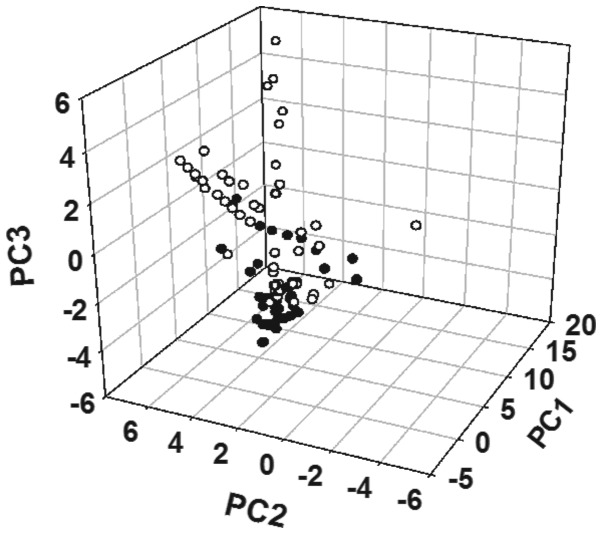
Principal component analysis of compounds from the chemical databases of *Salvia miltiorrhiza* (SM) and *Panax notoginseng* (PN). The black and white circles represent the former and the latter, respectively. PC1, first principal component; PC2, second principal component; PC3, third principal component.

Diversity descriptors (n=150) were used to map the chemical space of the SM and PN chemical databases using PCA (Fig. 2). A number of the key molecular descriptors of the compounds from the two databases are shown in Table II. The results were as follows: i) Considerable dispersion was observed in the first three principal components; ii) there was only a small overlap between the databases of the two molecules in chemical space; iii) the majority of the molecules did not violate ‘Lipinski’s rule of five’ (20).

### Comparison of the SM and PN chemical databases: Compounds with potential biological activity

To investigate whether the compounds in SM and PN were likely to be active in CVDs, the compounds with potential biological activity were predicted using virtual docking. The docking showed that various bioactive compounds in SM and PN targeted multiple proteins associated with CVDs. The average number of targets correlated with each compound in SM and PN were 5.0 and 3.6, respectively.

### Comparison of the SM and PN chemical databases: D-T network

**Figure 3.**
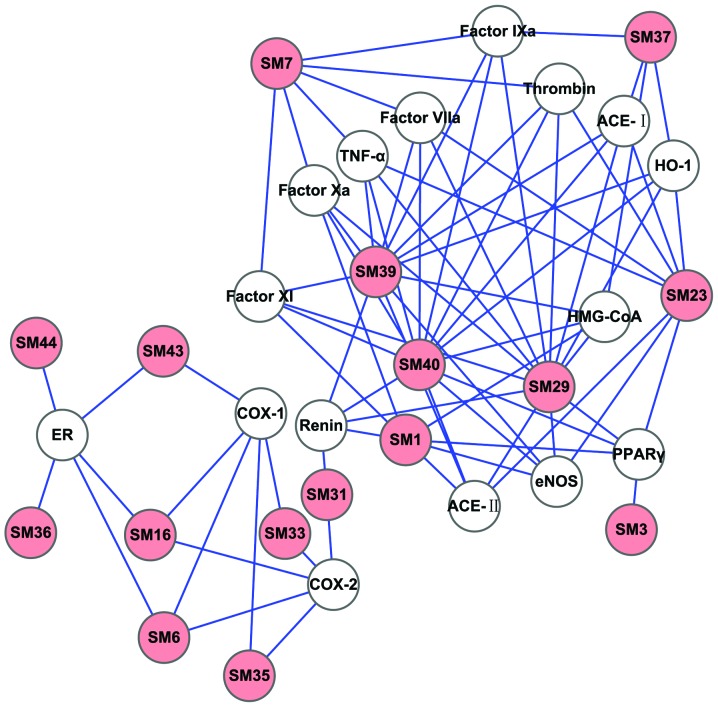
*Salvia miltiorrhiza* (SM) drug-target network. The white and pink circles represent target proteins associated with cardiovascular diseases (CVDs) and SM compounds, respectively.

**Figure 4.**
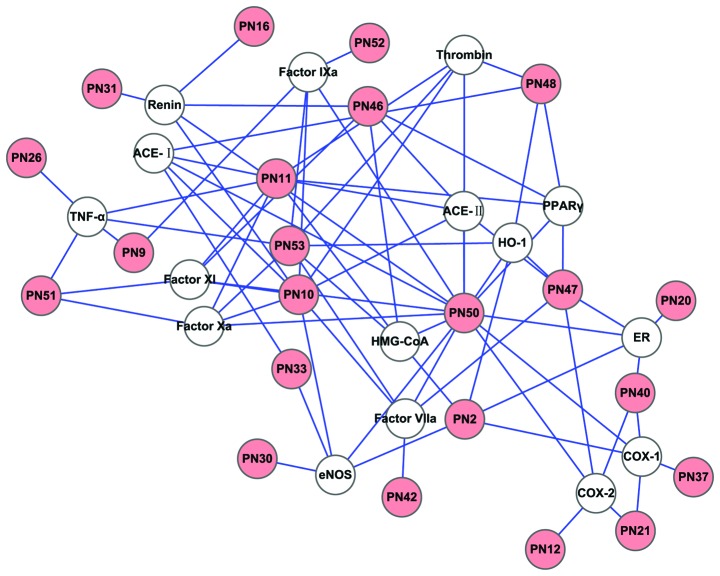
*Panax notoginseng* (PN) drug-target network. The white and pink represent target proteins associated with cardiovascular diseases (CVDs) and PN compounds, respectively.

**Figure 5.**
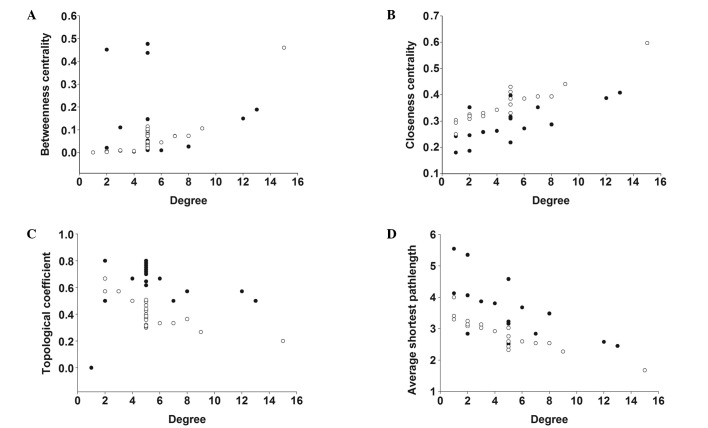
(A–D) Network analyses of *Salvia miltiorrhiza* (SM) and *Panax notoginseng* (PN) drug-target (D-T) networks. Parameter statistics of the SM D-T network are shown in black and the parameter statistics of the PN D-T network are shown in white.

**Figure 6.**
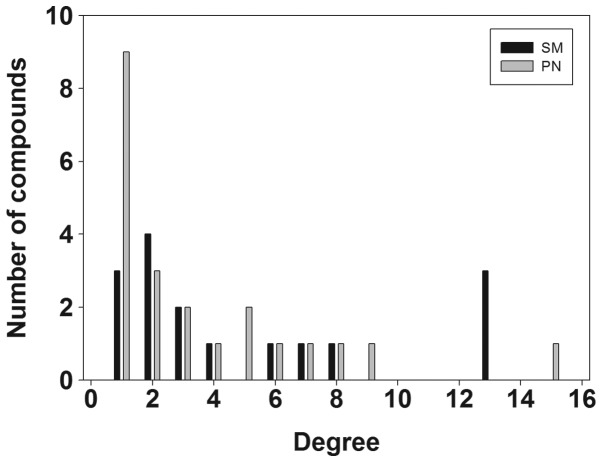
Distribution of the number of targets associated with each compound in *Salvia miltiorrhiza* (SM) and *Panax notoginseng* (PN).

The D-T network was generated by connecting the potential active compounds to their CVD-associated targets, in order to further clarify the associations between the potentially active compounds and their targets (Figs. 3 and 4). The simple parameters of the networks for SM and PN are shown in Table III. Plot parameters of these networks were used to characterize the global map of D-T interactions (Fig. 5) and the degree of compound nodes in the network was analyzed (Fig. 6). The key compounds with the top-five degrees in the two networks are shown in Table IV. These results demonstrated that SM and PN are able to act on multiple targets and exhibit different modes of action between compounds and targets.

## Discussion

CVDs are the leading cause of mortality in the world and pose a serious threat to human health. The diseases are complex and multifactorial and are caused by environmental, genetic and clinical risk factors (21). Therefore, CVDs tend to result from multiple molecular abnormalities. As a result, one drug acting on a single target may not lead to the effective treatment of the CVDs (22,23), and drug therapies addressing multiple targets have been receiving increasing focus.

For many years, numerous preparations of the herb pair comprising SM and PN have been used in the treatment of patients with CVDs (24). In the current study, a number of compounds in SM and PN, distributed among the different class groups, are shown in Fig. 1. The results reflect the structural diversity in the molecular composition of SM and PN and the differences between them. SM and PN were shown to possess a broad and different diversity in chemical space (Fig. 2), which indicates that they are likely to exert different effects (15). The statistics of the drug-like properties of the compounds from the SM and PN chemical databases (Table II) revealed the mean molecular weights to be 326.40 and 355.78, respectively; the mean number of hydrogen bond acceptors was 4.08 and 4.04, respectively; the mean number of hydrogen bond donors was 1.70 and 2.49, respectively and the mean AlogP was 3.57 and 4.34, respectively. According to the ‘rule of five’ (20), these compounds have desirable drug-like properties that make them suitable for use as oral drugs in humans. These results provided a good foundation for the screening of suitable active compounds.

A docking screening protocol was used to identify the compounds with multitarget potential for targets associated with CVDs using LigandFit. The docking results showed that the compounds in SM and PN exhibited potential biological activity with one or more target proteins. Among these compounds, 81.25% of the compounds in SM and 59.05% of the compounds in PN were able to act on more than one target protein. To further compare the effects of SM and PN on CVDs, we used the screening compounds and their interaction targets to generate a bipartite graph of drug-target interactions, in which a compound and a protein were connected to each other if the protein was an action target of the compound, giving rise to SM (Fig. 3) and PN (Fig. 4) D-T networks. The analyses of these networks (Fig. 5) showed minority common values of betweenness centrality, closeness centrality, topological coefficients and shortest path length, in addition to discriminating the detailed actions of SM and PN on CVDs. Zhu *et al* (25) proposed that identifying the common behavioral features from the network was likely to provide important information to enable the understanding of the drug-target interaction mechanisms in the human body; therefore, the topological analysis may reflect global knowledge concerning the particular properties of compounds and proteins involved in the network. The results indicated that SM and PN exhibited different modes of action. In addition, the compounds in SM and PN possessed different degrees in the model of the D-T network (Fig. 6 and Table IV). The majority of the compounds in Table IV have been described in previous studies (26–29). As mentioned previously, the herb pair consisting of SM and PN may possess a range of functions in the treatment of CVDs, via different compounds combining with different targets. Furthermore, the different modes of action of the pair may result in SM and PN exerting synergistic effects in CVDs. Zheng *et al* (7) demonstrated that SM primarily acted to expand blood vessels, while PN mainly participated in the protection of cardiac myocytes. Therefore, the combination of these two herbs improves coronary circulation and reduces the symptoms of myocardial ischemia. This may further corroborate the proposal that SM and PN are able to treat CVDs via different modes of action and that the combination of the two herbs is able to enhance the therapeutic effects.

In conclusion, the results of the present study demonstrated that: i) The compounds in SM and PN have diverse and drug-like properties; ii) the compounds in SM and PN have multitarget potential for targets associated with CVDs, and iii) the combination of SM and PN may enhance their activities in different potential multidrug combination therapies for CVDs. Furthermore, the method of computational pharmacology is able to intuitively trace out the different details of the structural classification, chemical space and modes of action of the compounds in SM and PN. This may be used as a new method for the identification of SM and PN, and to enable the improved understanding of herb pairs at the molecular level.

## Figures and Tables

**Table I.** Sixteen proteins associated with CVDs.

Protein	PDB code	Protein	PDB code
TNF-α	2AZ5	Factor IXa	1X7A
eNOS	1M9J	Factor Xa	1FJS
COX-1	1CQE	Factor VIIa	1YGC
COX-2	6COX	Factor XI	1ZSL
PPARγ	2HFP	HMG-CoA	1HW8
HO-1	3TGM	ACE I	1UZE
Thrombin	1YPJ	ACE II	1R4L
ER	1X7J	Renin	1BIL

CVDs, cardiovascular diseases; PDB, protein data bank; TNF-α, tumor necrosis factor-α; eNOS, endothelial nitric oxide synthase; COX, cyclooxygenase; PPARγ, peroxisome proliferator activated receptor γ; HO, heme oxygenase; ER, estrogen receptor; HMG-CoA, 3-hydroxy-3-methylglutaryl coenzyme A; ACE, angiotensin-converting enzyme.

**Table II.** Maximum, minimum and mean of the molecular descriptors of the SM and PN chemical databases.

Descriptors	SM			PN		
	Maximum	Minimum	Mean	Maximum	Minimum	Mean
Molecular weight	718.61	154.12	326.40	1271.44	118.18	355.78
No. of hydrogen acceptors	16	1	4.08	28	0	4.04
No. of hydrogen donors	9	0	1.70	18	0	2.49
AlogP	8.08	0.61	3.57	10.41	−4.54	4.34
No. of rotatable bonds	14	0	1.98	19	0	7.96
Molecular volume	438.35	88.49	219.33	853.72	86.43	270.14
Molecular surface area	652.14	148.91	313.64	1221.90	128.43	378.57
Molecular polar surface area	278.03	20.23	75.31	456.44	0	67.57

SM, *Salvia miltiorrhiza*; PM, *Panax notoginseng*.

**Table III.** Network properties of the SM and PN D-T networks.

Parameters	SM D-T network	PN D-T network
Network density	0.161	0.114
Network heterogeneity	0.596	0.652
Network centralization	0.275	0.308
Characteristic path length	3.544	2.762
Average no. of neighbors	5.000	4.211
Shortest paths	992 (100%)	1,406 (100%)

SM, *Salvia miltiorrhiza*; D-T, drug-target; PN, *Panax notoginseng*.

**Table IV.** Key compounds with the top-five degrees in the SM D-T network and PN D-T network.

SM D-T network			PN D-T network		
Index	Chemical name	Degree	Index	Chemical name	Degree
SM29	Monomethyl lithospermate	13	PN50	Quercetin	15
SM40	Salvianolic acid C	13	PN10	Dicapryl phthalate	9
SM39	Salvianolic acid A	12	PN11	Diisocapryl phthalate	8
SM23	Methyl rosmarinate	8	PN53	Stigmasterol	7
SM1	Baicalin	7	PN47	Panaxynol	6

SM, *Salvia miltiorrhiza*; D-T, drug-target; PN, *Panax notoginseng*.

## Abbreviations:

TCM: Traditional Chinese Medicine;
SM: *Salvia miltiorrhiza*;
PN: *Panax notoginseng* ;
CVDs: cardiovascular diseases;
TNF-α: tumor necrosis factor-α;
eNOS: endothelial nitric oxide synthase;
COX: cyclooxygenase;
HO: heme oxygenase;
PPARγ: peroxisome proliferator activated receptor γ;
ER: estrogen receptor;
HMG-CoA: 3-hydroxy-3-methylglutaryl coenzyme A;
ACE: angiotensin-converting enzyme;
D-T network: drug-target network
